# Concurrent Phacoemulsification and Encircling for Hypotony Maculopathy after Blunt Trauma

**DOI:** 10.1155/2020/6594170

**Published:** 2020-05-11

**Authors:** Takayuki Baba, Hirotaka Yokouchi, Shuichi Yamamoto

**Affiliations:** Department of Ophthalmology and Visual Science, Chiba University Graduate School of Medicine, Chiba, Japan

## Abstract

A 37-year-old Japanese man had his right eye hit by a fist. His right eye developed hypotony maculopathy and secondary cataract, and his visual acuity decreased to 20/200 with an intraocular pressure of 4 mmHg. He underwent phacoemulsification and aspiration, implantation of the intraocular lens, and encircling with a silicone tire. His visual acuity improved to 20/20 and stable for more than one year postoperatively. The intraocular pressure in his right eye increased to 12 mmHg, and maculopathy was resolved entirely. It was suggested that an encircling buckle obstructed the uveoscleral outflow through the cyclodialysis and increased intraocular pressure. Concurrent cataract surgery and encircling was sufficient to improve the vision.

## 1. Introduction

Blunt ocular trauma causes a wide variety of complications in the injured eye [1]. The hypotony maculopathy develops when the intraocular pressure (IOP) becomes low due to the angle recession and the cyclodialysis [2]. The young patients and myopic patients tend to develop hypotony maculopathy [3]. The hypotony sometimes recovers spontaneously, but the surgical intervention is recommended for the cases with persistent hypotony because the long-standing maculopathy damages the macula [4]. The treatment options for hypotony maculopathy are as follows: medical treatments including topical atropine [5], laser treatment of angle [6], cryotherapy [7], suturing ciliary body [8], intraocular injection of expanding gas [9], implantation of intraocular lens at sulcus [10], vitrectomy [11], and encircling buckle [12, 13].

We treated a case of hypotony maculopathy and secondary cataract after blunt trauma by encircling buckling and phacoemulsification and implantation of the intraocular lens. The combination of two surgeries is not common, but we could achieve good anatomical and functional recovery after the initial surgery.

## 2. Case Report

**Figure 1:**
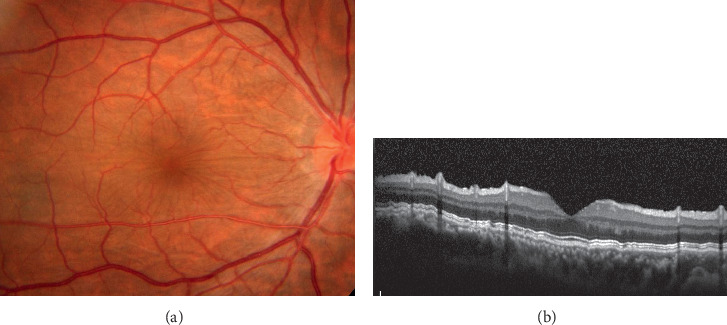
Fundus photographs and optical coherence tomographic images in a patient with hypotonic maculopathy after blunt ocular trauma. (a) Choroidal and retinal folds were observed at the posterior pole. The patient's visual acuity was 20/50, with an intraocular pressure of 5 mmHg. (b) An image of vertical optical coherence tomography through the fovea showed significant choroidal and retinal folds.

**Figure 2:**
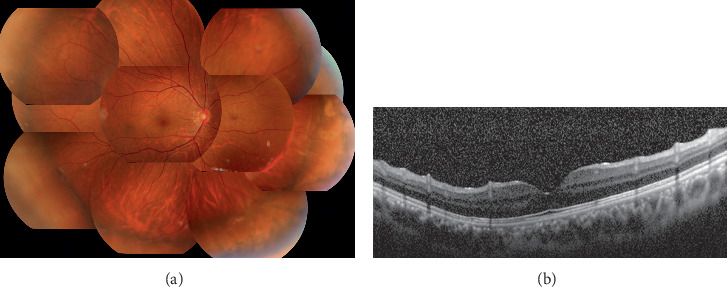
Postoperative images after encircling buckle and cataract surgery at one year after the surgery. (a) A fundus photograph at one year after the surgery. The protrusion by encircling was observed. His visual acuity improved to 20/20 with an intraocular pressure of 12 mmHg. (b) A vertical optical coherence tomography image through the fovea. The retinal and choroidal folds resolved completely.

A 37-year-old Japanese man had his right eye hit by a fist in an accident. He felt visual impairment in his right eye and was transferred to the emergency unit of Chiba University Hospital. His visual acuity was 20/200 in the right eye and 20/16 in the left eye at the initial presentation. The refractive error of spherical equivalent was −12.0 diopters in his right eye and −4.0 diopters in his left eye. He had a laceration of the lower eyelid, hyphema, and angle recession for 180 degrees and commotio retinae. The ultrasound biomicroscopy presented a cyclodialysis cleft in the nasal half. The intraocular pressure was 5 mmHg in the right eye and 15 mmHg in the left eye. He started topical steroids and atropine. Although the inflammation gradually decreased, the IOP in the right eye remained at a low level. There were significant findings of hypotony maculopathy with retinal and choroidal folds at macular, and his visual acuity was 20/50 (Figure 1). At 11 months after the trauma, his visual acuity decreased to 20/200 because of the progression of cataract concomitant with maculopathy. He underwent phacoemulsification and aspiration, implantation of the intraocular lens, and encircling with a 7 mm width silicone tire to increase IOP by blocking the suprachoroidal outflow. There was no complication during the surgery. His visual acuity improved to 20/20 with a refractive error of −6.0 diopters at one month after the surgery. The intraocular pressure in his right eye increased to 12 mmHg, and the retinal and choroidal folds were completely resolved at 3 months postoperatively (Figure 2). The visual acuity and intraocular pressure were stable for 7 years after the surgery.

## 3. Discussion

Our case with hypotony maculopathy showed good response to the combination of surgery, encircling buckle and phacoemulsification and aspiration with intraocular lens implantation. The encircling buckle is thought to exert the effect because the buckle reduces the uveoscleral outflow at 360 degrees. The encircling buckle is useful, especially for the cases with a wide area of angle recession and cyclodialysis [14]. For these cases with an extensive detachment of ciliary body, the multiple ciliary sutures which are challenging with a risk of complications such as intraocular hemorrhage and creating another cyclodialysis cleft [15] are required if the direct cyclopexy is attempted. The less invasive treatments, such as laser and cryotherapy, are usually not effective for these cases with a wide recession.

We performed cataract surgery in the same session. The concomitant cataract surgery benefits the improvement of vision right after the surgery. The problem of concomitant cataract surgery is the difficulty of the calculation of IOL. The axial length measurement becomes shorter in a hypotonic eye [16] and becomes longer after the encircling buckle in short [17] and long [18] term. In our case, the postoperative spherical equivalent showed a little myopic shift, but myopic change is more tolerable for patients than the hyperopia.

Finally, we could successfully treat a case with hypotony maculopathy by a combination of encircling buckle and cataract surgery. This technique is useful for cases with a wide area of angle recession and cataract formation. The risk of intraoperative complications such as intraocular hemorrhage seemed less for this method.
